# Idiopathic gangrene of the tongue: a case report

**DOI:** 10.4076/1757-1626-2-6290

**Published:** 2009-06-22

**Authors:** Anand Pandey, Lokesh Saluja, Ajay Agarwal, Sudesh K Sagar

**Affiliations:** Department of Surgery, Sri Ram Murti Smarak Institute of Medical SciencesBareilly, Uttar PradeshIndia

## Abstract

**Introduction:**

Gangrene of the tongue is an extremely rare condition. We encountered a 35-year-old patient who presented with this entity.

**Case presentation:**

A 35-year-old male patient from rural background presented with blackening of the tongue. Its exact cause could not be ascertained. Examination revealed it to be gangrene. It was treated with excision of the gangrenous part.

**Conclusion:**

As this is a very rare condition, it is being reported with a brief review of the relevant literature. The treatment must be based on the etiology; however, if the etiology is not known, it must be symptomatic.

## Introduction

The tongue is an important part of oral cavity, which helps in taste, salivation, and deglutition. Gangrene of the tongue is an extremely rare phenomenon because of its excellent blood supply. The chief blood supply to the tongue is by the lingual artery. Besides this, the ascending pharyngeal artery and the external facial artery also contribute deep branches to the tongue [1].

We encountered an extremely rare case of gangrene of the oral tongue, the exact etiology of which could not be ascertained. Being a rare entity, it is being reported with a brief review of the relevant literature.

## Case presentation

A 35-year-old male of Indian origin was admitted in the casualty with complaint of blackening of the oral tongue for last three days. The patient was mentally retarded and hence, the exact history could not be ascertained. The attendants noticed a foul smell from the mouth of the patient, and on seeing the black tongue they were horrified. For this reason, they brought the patient to the hospital. There was doubtful history of repeated tongue bite by the patient himself, but there was no history of oral bleeding. There was no history of any other significant associated illness.

**Figure 1.**
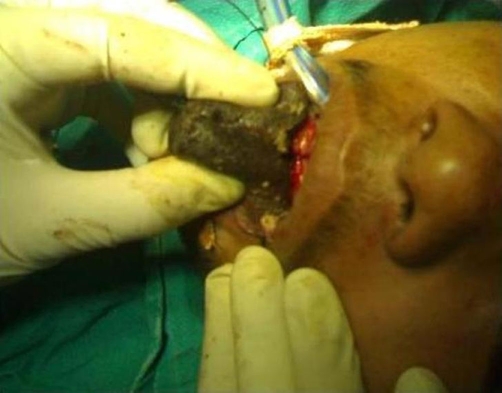
Gangrenous tongue, seen at the time of surgery. It was foul smelling.

On examination, the pulse rate was 78/minute and respiratory rate was 15/minute. The general condition of the patient was satisfactory. On examining the oral cavity, anterior two-third of the tongue was gangrenous (Figure 1). There was foul smell emanating from the mouth.

**Figure 2.**
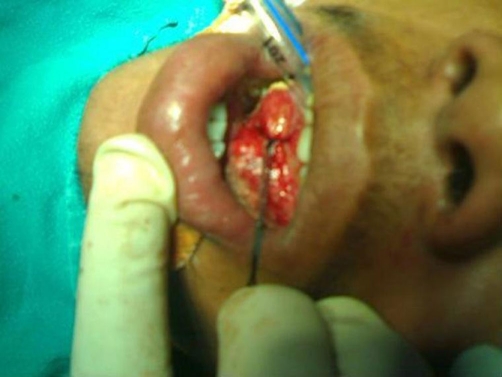
Postoperative view, after excision of the gangrenous part. A thread is tied to prevent tongue fall in the postoperative period.

The patient was shifted to the operation theatre, where resection of the gangrenous tongue was done. The remaining tongue was repaired (Figure 2). The postoperative period was uneventful. The patient was discharged in a satisfactory condition on seventh postoperative day.

Histopathology of the specimen was non-specific. It reported necrosis in the specimen with specific etiology for it.

## Discussion

The circulatory disturbances of the tongue are extremely rare because of its rich blood supply [2]. Lingual artery, ascending pharyngeal artery and external facial artery form a good network that supplies the tongue; hence, gangrene of the tongue is unlikely [1].

Gangrene of the tongue is a rare phenomenon, less than 30 cases have been reported in the literature. An important cause of gangrene of the tongue is giant cell or temporal arteritis [3-5]. This form of gangrene can be confirmed by the histopathology report. As histopathology of the specimen was non-specific in our patient, this was ruled out to be the cause.

Other reported causes of the gangrene are intra-aortic balloon pump for cardiogenic shock [6], self-application of an elastic rubber band [7], atherosclerotic arterial occlusion [1]. These have been reported merely as sporadic case reports. None of these could be a possibility in our patient.

As the patient was mentally retarded, we were unable to glean the exact history. Based on the history of self-inflicted trauma to the tongue, we speculate that it may be the cause for the gangrene to occur. There may be impairment to the venous drainage, which appears to develop only as a consequence of an extensive posttraumatic or inflammatory edema of the floor of the mouth and tongue base [2]. This impairment of venous drainage may result in a large acute swelling of the tongue, or to ischemia carrying a painful tongue swelling, and possibly tongue necrosis [2]. However, we do agree that exact etiology was not known.

To conclude, gangrene of the tongue is a rare phenomenon. The treatment must be based on the etiology; however, if the etiology is not known, it must be symptomatic.
